# Effect of Simulated Microgravity on Human Brain Gray Matter and White Matter – Evidence from MRI

**DOI:** 10.1371/journal.pone.0135835

**Published:** 2015-08-13

**Authors:** Ke Li, Xiaojuan Guo, Zhen Jin, Xin Ouyang, Yawei Zeng, Jinsheng Feng, Yu Wang, Li Yao, Lin Ma

**Affiliations:** 1 Department of Radiology, Chinese PLA General Hospital, Beijing, China; 2 Magnetic Resonance Center, 306 Hospital of PLA, Beijing, China; 3 College of Information Science and Technology, Beijing Normal University, Beijing, China; 4 The Third Laboratory, China Astronaut Research and Training Centre, Beijing, China; 5 Outpatient Department of 61599 Unit of PLA, Beijing, China; Chinese Academy of Sciences, CHINA

## Abstract

**Background:**

There is limited and inconclusive evidence that space environment, especially microgravity condition, may affect microstructure of human brain. This experiment hypothesized that there would be modifications in gray matter (GM) and white matter (WM) of the brain due to microgravity.

**Method:**

Eighteen male volunteers were recruited and fourteen volunteers underwent -6° head-down bed rest (HDBR) for 30 days simulated microgravity. High-resolution brain anatomical imaging data and diffusion tensor imaging images were collected on a 3T MR system before and after HDBR. We applied voxel-based morphometry and tract-based spatial statistics analysis to investigate the structural changes in GM and WM of brain.

**Results:**

We observed significant decreases of GM volume in the bilateral frontal lobes, temporal poles, parahippocampal gyrus, insula and right hippocampus, and increases of GM volume in the vermis, bilateral paracentral lobule, right precuneus gyrus, left precentral gyrus and left postcentral gyrus after HDBR. Fractional anisotropy (FA) changes were also observed in multiple WM tracts.

**Conclusion:**

These regions showing GM changes are closely associated with the functional domains of performance, locomotion, learning, memory and coordination. Regional WM alterations may be related to brain function decline and adaption. Our findings provide the neuroanatomical evidence of brain dysfunction or plasticity in microgravity condition and a deeper insight into the cerebral mechanisms in microgravity condition.

## Introduction

Over the last two decades, there have been a lot of long-duration spaceflights. Studies have shown that astronauts’ cognitive and behavioral activities would be impaired in space environment where there are some harmful factors, such as microgravity, radiation, noise and changed circadian rhythm [1–4]. Microgravity is the main cause of degradation performance, which may bring potential risks in space exploration [5,6]. Meanwhile, the adaptation or plasticity may occur in central nervous system (CNS) during long spaceflight. Therefore, it is of great significance to reduce the odds of accident and find out the mechanisms of physiological changes in CNS under the microgravity influence.

The cerebral effects of microgravity have been a persistent concern. Physiology experiments have successfully observed changes in volumes of cerebrospinal fluid, cerebral blood flow and intracranial pressure caused by the redistribution of an astronaut’s body fluid toward head in a weightless environment [7–9]. These changes in turn may lead to structural remodelling and alter cerebral autoregulation [10]. A study conducted with rats showed that cerebral arteries hypertrophy occurred in response to microgravity simulated by tail-suspension [11] and blood vessel remodeling occurred with increased vessel wall thickness and vessel diameter in the brain vasculature after two weeks of head-down tilt on earth [12,13]. Additionally, cerebral vasoconstriction may increase following long-term space flight [14], not similar to that seen in hindquarter arteries [15].

On the other hand, previous studies demonstrated effects of microgravity on neurotransmitter concentrations and neural architecture plasticity on the cerebra in animal models [16–18], such as in the somatosensory cortex and the cerebellum with a decreased number of synapses and degeneration of axonal terminals after microgravity exposure [19–22]. However, Ross demonstrated an increased synapse number of hair cells in the rat utricular macula, mammalian gravity receptors in the vestibular system, by approximately 41% to 55% exposed to microgravity [23,24]. A recent study observed that alterations in afferent signaling, as a result of lack of lower extremity weight bearing, would induce cortical reorganization by altering corticospinal excitability plasticity in a bed rest model [25]. All these studies suggested that microstructural alterations could occur in multiple brain regions as a result of the interaction between exposure to microgravity and spaceflight [26]. But, whether and to what extent the brain functional changes are related to microgravity induced brain structural changes, apart from vestibular reorganization, or more peripheral changes such as bodily fluid shifts and muscle unloading, is not yet known [19,20,23].

To the best of our knowledge, no study so far was conducted to investigate the adaptive responses of human brain structure that contribute to the observed performance degradations in microgravity. In this context, we performed this study to investigate structural modifications in brain with 14 volunteers on simulated microgravity for 30 days examined by magnetic resonance imaging (MRI). We used -6° head-down bed rest (HDBR), a method which has proved its usefulness as a reliable simulation model for the most physiological effects of spaceflight [27–30], to simulate microgravity. Given that numerous studies have demonstrated cortical plasticity in response to various environmental alterations, such as microstructural alterations in GM and WM of multiple brain regions, has been shown in chronic hypoxia-exposed high-altitude residents [31–33], we hypothesize that there would be alternations in GM and WM of the brain due to microgravity.

## Materials and Methods

### Subjects

Eighteen healthy male volunteers were recruited through the bed rest facility located at Astronaut Research Training Center (Beijing, China) and underwent -6° head-down bed rest (HDBR) to simulated microgravity condition for 30 days. Subjects were 22–36 years old and all passed conventional physical examination. All subjects were right-handed, non-smokers, with normal body weight and body mass index, and had no documented neurological disorders or history of head injuries with loss of consciousness. All subjects were participating in several other studies which did not affect one another. The experimental protocol was approved by the Ethical Committee of Astronaut Research Training Center. Before the experiment, all subjects signed informed consent forms, which were kept in archive. After the experiment, subjects were compensated for participation. Physiological data of all subjects were recorded before and after HDBR.

### Design

This is a self-controlled study investigating the effects of microgravity on brain. Because extended exposure to a head-down tilt position can duplicate many effects of a low-gravity environment, -6° head-down bed rest (HDBR) was used to simulate microgravity condition. Bed rest subjects remained in bed with their heads tilted down for 30 consecutive days. During the HDBR, they were supplied with adequate water and food, and allowed to read and watch TV. But their heads were prevented from moving from the bed to keep the redistribution of body fluid toward head. To prevent muscle wasting after long-term bed rest, all subjects were required to do adequate lower limb exercise every day.

### MRI Data Acquisition

Images were acquired on a Siemens Verio 3.0T MR scanner (Erlangen, Germany) before and after HDBR. A 3D structural MRI was acquired using a T1-weighted MPRAGE sequence (TR/TE = 1900 ms/2.19 ms, FOV = 250×219 mm^2^, NEX = 1, matrix = 256×246, slice thickness = 1.0 mm, and 176 sagittal slices in the third dimension) covering the entire brain. Conventional axial T2 images were also acquired. A DTI pulse sequence with single shot diffusion-weighted echo planar imaging (TR/TE = 3600/95 ms, FOV = 230×230 mm^2^, NEX = 1, matrix = 128×128, slice thickness = 4 mm) was applied sequentially in 30 non-collinear directions (b-value = 1000 s/mm^2^) with one scan. We acquired 50 contiguous slices covering the entire brain.

### VBM Analysis

The preprocessing of structural MRI data was performed using the VBM8 Toolbox (http://dbm.neuro.uni-jena.de/vbm8) implemented in SPM8 (Statistical Parametric Mapping, http://www.fil.ion.ucl.ac.uk/spm). The main steps of preprocessing include intra-subject realignment, bias correction, segmentation and normalization. Firstly, each subject’s follow-up scan was realigned to the corresponding baseline scan to calculate a mean image used as the reference template, and the above two scans were realigned to this template. Secondly, bias correction was done to correct the signal inhomogeneities of the realigned images. Thirdly, for all subjects, the bias-corrected images were segmented into GM images using adaptive Maximum A Posterior (MAP) [34] and partial volume estimation (PVE) [35]. Fourthly, a newly developed method, Diffeomorphic Anatomical Registration using Exponential Lie Algebra (DARTEL) [36,37], was applied to normalize the mean image and the normalization parameters were estimated, then the normalization was performed on the GM images using those parameters. Thereafter, the normalized GM partitions were multiplied by the Jacobian determinants from the deformations to preserve the total amount of tissue in the native spaces. Finally, the GM maps for all subjects were smoothed with an 8-mm full width at half maximum (FWHM) Gaussian kernel and entered into the subsequent statistical procedure.

Paired t-test was used to determine the differences between baseline GM maps and the corresponding follow-up maps. The statistical significance level was set at P < 0.05 (false discovery rate (FDR) corrected for multiple comparisons).

### TBSS Analysis

DTI data were processed using the FSL software (FMRIB Software Library 4.1.9, http://www.fmrib.ox.ac.uk/fsl). Eddy current correction and head motion were implemented using the FMRIB’s Diffusion Toolbox (FDT) 2.0 [38] on the raw DTI data. Secondly, the fractional anisotropy (FA) maps were generated based on the diffusion tensor.

Then, all subjects’ FA images were processed using Tract-Based Spatial Statistics (TBSS) by the following steps: 1) Nonlinear registration was applied to each subject’s FA images to find the most representative image as the target image. 2) Then the target image was normalized to Montreal Neurological Institute (MNI) standard space. 3) Each subject’s FA images were nonlinear registration to the target and then normalized to the MNI space. 4) The mean FA image and mean FA skeleton were created. 5) All the FA images were projected to the mean FA skeleton to obtain their corresponding skeletonised images.

The voxelwise statistics were performed using the randomized permutation test with a General Linear Model (GLM) of paired two-group difference (baseline scans and follow-up scans) for all subjects’ skeletonised FA images. We set the mean FA skeleton’s threshold value to 0.2 and the number of permutation to 5000. The statistical significance level was set at P < 0.01 without multiple-comparison correction.

## Results

### Demographic and Physiological Data

Eighteen male healthy volunteers who met the inclusion and exclusion criteria received MRI scans. Four cases were discarded because of interruption of HDBR. Finally, the data of fourteen subjects were used for this study. Descriptive characteristics and physiological data were shown in Table 1. No significant changes in weight and blood pressure were observed before and after HDBR (p > 0.05).

**Table 1:** Demographic and physiological characteristics of subjects.

	Before HDBR	After HDBR	P
**Number of subjects**	14	14	
**Age (years) (mean**±**SD)**	28.8±4.7(22–36)	28.8±4.7(22–36)	
**Height**	171.9±4.3(165–178)	171.9±4.3(165–178)	
**Weight**	66.8±7.1(58–84)	68.0±6.5(61–83)	0.041
**Systolic blood pressure(mmHg)**	119.4±8.1(100–128)	124.1±7.5(102–132)	0.017
**Diastolic blood pressure(mmHg)**	77.7±8.0(70–88)	76.1±5.3(70–88)	0.429

### Changes in GM volume before and after HDBR

**Fig 1:**
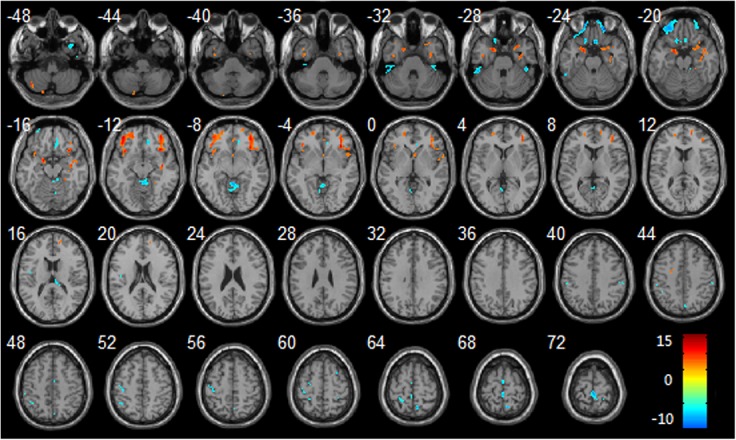
Regional changes of GM volumes after HDBR as revealed by voxel-based morphometry. Three-dimensional slices depicting regions showing decreased GM volume (red) in the bilateral frontal lobes, parahippocampal gyrus, insula, right temporal pole, right hippocampus and increased GM volume (blue) in vermis, bilateral paracentral lobule, right precuneus gyrus, left precentral gyrus, left postcentral gyrus overlaid on a T1-weighted MRI anatomical image in the stereotactic space of the Talairach template.

GM volume reduction was found in the bilateral frontal lobes (inferior frontal gyrus, middle frontal gyrus, superior frontal gyrus), insula, parahippocampal gyrus and right temporal pole, hippocampus after HDBR (Table 2); among them, bilateral frontal lobes were the most remarkable area. In contrast, GM volume increase was found in the vermis, left parietal lobe (posterior central gyrus, inferior parietal gyrus), precentral gyrus and right paracentral lobule, precuneus after HDBR (Table 3); among them, vermis was the most remarkable area. Regions of GM volume changes were overlaid on a T1-weighted MRI anatomical image in the stereotactic space of the Talairach template (Fig 1).

**Table 2:** Regional information of decreased GM volume after HDBR.

Areas	Volume (mm^3^)	Brodmann areas	MNI coordinate (mm)			t-score (peak)
			X	Y	Z	
**Frontal_Inf_Orb_R**	2400	38	36	21	-14	15.36
**Frontal_Inf_Orb_L**	1711	11	-38	47	-12	14.08
**Frontal_Mid_Orb_L**	1235	11	-38	47	-11	14.75
**Frontal_Mid_Orb_R**	1023	47	38	48	-11	11.13
**Temporal_Pole_Sup_R**	827	36	30	11	-29	8.82
**Frontal_Mid_R**	817	47	39	41	9	11.77
**ParaHippocampal_L**	756	28	-17	0	-18	10.62
**Insula_L**	668	38	-33	18	-12	10.97
**Frontal_Sup_Orb_L**	665	11	-20	48	-12	8.93
**Insula_R**	635	47	36	27	-5	15.74
**ParaHippocampal_R**	618	20	35	-18	-20	9.1
**Hippocampus_R**	442	20	39	-12	-14	12.6

**Table 3:** Regional information of increased GM volume after HDBR.

Areas	Volume (mm^3^)	Brodmann areas	MNI coordinate (mm)			t-score (peak)
			X	Y	Z	
**Vermis_4_5i**	712		3	-47	-8	-10.42
**Rectus_R**	658	11	6	20	-26	-10.77
**Paracentral_lobule_R**	651	4	2	-32	71	-12
**Frontal_Sup_Orb_L**	574	11	-21	42	-21	-9.91
**Parietal_Inf_L**	446	40	-41	-51	51	-9.06
**Precuneus_R**	415	5	9	-59	66	-10.23
**Precentral_Li**	415	6	-41	-21	54	-8.39
**Vermis_3i**	412		5	-47	-9	-10.24
**Temporal_Inf_L**	405	20	-51	-41	-29	-10.06
**Postcentral_L**	388	4	-39	-24	51	-9.22

### Changes in FA before and after HDBR

**Fig 2:**
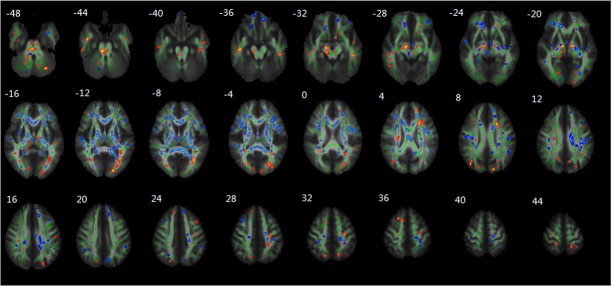
Regional changes of FA values as revealed by TBSS after HDBR. The group’s mean FA skeleton (green) was overlaid on the mean FA images. The threshold of mean FA skeleton was set at 0.2; the regions with decreased FA after HDBR are red coloured, and the regions with increased FA after HDBR are blue coloured.

After HDBR, subjects showed significant decrease in FA values in multiple regions, including bilateral WM tracts in frontal lobe, temporal lobe, parietal lobe, occipital lobe, thalamus, brainstem and cerebellum. In contrast, subjects showed significant increase in FA in multiple WM regions, including bilateral WM tracts in frontal lobe, temporal lobe, parietal lobe, occipital lobe, thalamus, forceps major of corpus callosum, cingulum, internal and external capsule and right brainstem, cerebellum (Fig 2).

## Discussion

To our knowledge, this study is the first global assessment of the effects of microgravity on human brain structure by using MR techniques. By using a combined analysis method of VBM and TBSS, we found that microstructures of GM and WM were changed after 30 days of HDBR. The findings give new insight into the underlying neural mechanisms of spaceflight-induced changes in brain structure.

Our study showed GM volume reduction in multiple brain regions mostly located in the bilateral frontal lobes, temporal poles, insula, parahippocampal gyrus and right hippocampus in subjects after HDBR. In contrast, GM volume increase was found in the vermis, bilateral paracentral lobule, right precuneus gyrus, left precentral gyrus and left postcentral gyrus after HDBR. Accroding to neuroanatomy, these regions showing GM changes are closely associated with the functional domains of performance, locomotion, learning, memory and coordination. Actually, similar brain regions have been shown to be involved in neurological dysfunctions [39–41]. Furthermore, our results are also compatible with the relevant findings of the previous space medical studies, and provide the anatomical evidences for sensorimotor and cognitive dysfunction in spaceflight [42–44].

On the microscopic structure, GM was composed of neurons, nerve fibers, glial cells and capillaries. According to Zatorre et al. [45], candidate mechanisms for GM changes included synaptogenesis, gliogenesis, neurogenesis and vascular changes. DeFelipe et al. [46] reported that development in microgravity led to changes in the number and morphology of cortical synapses in a laminar-specific manner in rats, and the result indicated that terrestrial gravity is a necessary environmental parameter for normal cortical synaptogenesis. We speculate that GM volume decrease in simulated microgravity may result from decreased neurons impulse and suppressed synaptogenesis when activities are restricted in HDBR. Changes of cerebral vascular flow and increased vasoconstriction as a result of microgravity exposure may also contribute to GM volume decrease [13,47]. Whereas, several studies have suggested that GM volume increase was associated with neurogenesis. For example, Kwon et al. [48] reported exercise and bright light could improve memory and promote hippocampal neurogenesis in adult rats. Leavitt et al. [49] reported that aerobic exercise could increase hippocampal volume and improve memory in patients with multiple sclerosis. In our study, regions showing increased GM volume were concentrated in motor function areas, such as vermis and paracentral lobule. We speculate that it was induced by sensorimotor adaptation or compensatory processing in simulated microgravity.

On the other hand, we observed decrease of FA value in multiple WM regions, including the bilateral WM tracts in frontal lobe, temporal lobe, parietal lobe, occipital lobe, thalamus, brainstem and cerebellum after HDBR. According to Renoux et al. [50], decreased FA was linked with an increase of the extracellular space (dysmyelination, axonal loss, unpacking of WM fibers, and so forth) as well as a decrease of the intracellular space (edema) in the WM. It has been reported that DTI with FA computation is more sensitive than conventional MR to detect integrity of WM. For example, decreased FA is one of the earliest MRI abnormalities observed in cognitively normal individuals who are at an increased risk for AD [51]. Decreased FA at early stages of MCI and AD could predict the decline in cognitive function [52]. So we think that impairment of WM occurred in simulated microgravity condition, and it may be related to the sensorimotor and cognitive function decline.

For the past decade, most studies have reported decreased FA in WM, but contrary results were also reported recently, especially in skill training studies [53,54]. Furthermore, wildly increased FA has also been reported in obsessive compulsive disorder patients, including in the regions of cingulum and internal capsule [55], and corpus callosum [56]. These studies have suggested that the higher FA might be related to an increase in the connectivity of WM bundles. In our study, we observed widely increased FA values in WM after HDBR, especially in the regions of forceps major of corpus callosum, cingulum, internal and external capsule. We speculated that higher FA in these regions might reflect strengthened connectivity in microgravity condition, and it may occur as a compensatory response to brain function decline [57] or fibre reorganization [58].

A few limitations in our present study should be noted. The first is that MR data of subject was acquired at only two time points. In the future, MR data also should be acquired in bed rest and 1 week after bed rest, which would help reveal the mechanisms of brain morphological change under simulated microgravity. Furthermore, to evaluate the stability and reliability of MRI measures over time, we should run a parallel study with ground-based control participants testing across multiple time points. The second is that there were no female subjects, so the results of our study can not tell the actual situation of females. Thirdly, we present the results of TBSS without multiple-comparison correction, because the change of WM under microgravity was too subtle to be discovered yet. Considering the lack of control may result the false positive errors, so the results should be treated with caution and more research is needed to confirm the results.

In conclusion, our study demonstrated regional GM and WM alterations in subjects who underwent HDBR for 30 days. Regional GM alterations are closely related to the functional domains of performance, locomotion, learning, memory and coordination. Regional WM alterations may be related to brain function decline and adaptation. Our findings provide the neuroanatomical evidence of brain dysfunction or plasticity in microgravity condition and a deeper insight into the cerebral mechanisms in microgravity condition.
